# Single Fraction Stereotactic Radiosurgery for Retreatment of Skull Base Recurrent Head and Neck Malignancies

**DOI:** 10.7759/cureus.1206

**Published:** 2017-05-01

**Authors:** Rajal A Patel, Derrick Lock, Thomas Kim, Sandeep Samant, James P Chandler, Bharat B Mittal, Tim J Kruser

**Affiliations:** 1 Department of Radiation Oncology, Northwestern University Feinberg School of Medicine, Chicago, IL, USA; 2 Chicago Medical School, Rosalind Franklin University of Medicine and Science; 3 Department of Otolaryngology Head and Neck Surgery, Northwestern University Feinberg School of Medicine, Chicago, IL, USA; 4 Department of Neurological Surgery, Northwestern University Feinberg School of Medicine, Chicago, IL, USA

**Keywords:** stereotactic radiosurgery, head and neck malignancy, recurrence, skull base, reirradiation

## Abstract

**Introduction:**

Recurrent head and neck carcinomas are notoriously difficult to treat. Salvage surgery, brachytherapy, and repeat external beam radiotherapy have all been utilized, achieving modest local control at the expense of elevated toxicity. We performed a retrospective review to evaluate the efficacy of single fraction stereotactic radiosurgery (SRS) for the treatment of recurrent head and neck carcinomas.

**Methods:**

Eighteen previously irradiated patients diagnosed with a locoregionally recurrent head and neck malignancy and treated with single fraction SRS from 2000 to 2016 were analyzed. Actuarial rates for local control (LC) and overall survival (OS) were calculated with Kaplan-Meier estimates.

**Results:**

Median follow-up was 16.1 months and SRS dose was 13.3 Gy. One-year rate of LC was 52.7% (95% confidence interval [CI] 29%-72%). Median OS was 25.4 months. Parotid gland primary had an increased risk of progressive disease (PD) following SRS (hazard ratio [HR] 4.24, p=0.02). Squamous cell histology was negatively associated with OS (HR 3.85, p=0.03). One patient experienced grade 2 radionecrosis.

**Conclusions:**

Single fraction SRS is an acceptable treatment for previously irradiated patients with recurrent head and neck primary malignancies. Dose escalation to optimize LC should be examined.

## Introduction

The treatment for locally advanced head and neck malignancies has evolved over the last several decades to entail a multi-modality approach. The use of definitive concurrent chemoradiation (CRT), along with postoperative CRT for high-risk patients, has improved both local control (LC) and survival rates [1-2]. However, obtaining LC while minimizing toxicity remains the greatest hurdle for treating head and neck malignancies, with many patients experiencing a locoregional failure [3-4]. Salvage surgery for recurrent head and neck squamous cell carcinomas after high dose radiotherapy carries substantial risk of morbidity and mortality and often may not be possible given the extent of recurrent disease [5]. Similarly, salvage external beam radiotherapy is fraught with risk considering that adjacent critical structures commonly approach normal tissue tolerance parameters during the initial radiation course [6].

Agents such as cetuximab and nivolumab have recently demonstrated improvement in both progression-free survival (PFS) and overall survival (OS) for patients with recurrent or metastatic head and neck carcinomas [7-8]. Unfortunately, local response rates with these agents remain low. Additional locoregional therapies continue to warrant consideration to maintain LC as patients with recurrent disease are living longer.

In addition to conventional external beam radiation, stereotactic radiosurgery (SRS) has previously been implemented in the treatment of primary head and neck malignancies. When used during primary treatment, SRS as a boost can improve LC in primary nasopharyngeal carcinomas [9]. Fractionated SRS has also been employed for patients with recurrent head and neck malignancies with reports demonstrating complete response rates of 40%-60% and adequate short-term control [10-12]. SRS for patients with recurrent disease permits sparing of radiosensitive tissues, which limits morbidity while delivering an appropriate dose to potentially achieve durable LC. With this in mind, we conducted a review of patients with recurrent head and neck primary malignancies to examine outcomes associated with single fraction SRS.

## Materials and methods

### Patients

After getting approval from the Institutional Review Board Office of Northwestern University for study STU00202784, patients that were diagnosed with a locoregionally recurrent head and neck primary malignancy and treated with single fraction SRS from 2000 to 2016 were retrospectively reviewed. Patients were excluded if their recurrence was deemed to be the result of hematogenous metastasis and not direct extension or perineural spread. Ultimately, 18 patients were identified and included in this review.

### Radiosurgery

The Leskell Gamma Knife 4c® (Elekta; Stockholm, Sweden) treatment system was used from 2000 to 2009, and the Leskell Gamma Knife Perfexion® treatment system (Elekta; Stockholm, Sweden) was used from 2009 to 2016 to treat all patients. A contrast or double contrast MRI was obtained the morning of SRS treatment for each patient. The gross tumor volume (GTV) constituted all gross disease seen on imaging and exam. No additional margin was added for a clinical target volume (CTV) or for a planning target volume (PTV). The treatment volume was reviewed by both a radiation oncologist and either a neurosurgeon or otolaryngologist prior to dose determination and patient treatment. All treatment doses were prescribed to the 50% isodose line and delivered in a single fraction.

### Follow-up

All patients were scheduled to receive an MRI or CT scan two to three months following SRS. Every patient then underwent serial imaging and a physical exam with a flexible fiberoptic nasolaryngoscopy every three to six months until lost to follow-up or clinically indicated.

### Statistical analysis

Time to event analyses were measured from the date of SRS. LC was defined as a complete response, partial response, or stable MRI imaging per response evaluation criteria in solid tumors (RECIST) on follow-up scans. Progressive disease (PD) following SRS on any follow-up scan was further stratified into in-field, if >95% of the disease was within the treatment field; marginal, if 20-95% of the disease was within the treatment field; or out-field, if <20% of the disease was within the treatment field [13]. PD was backdated to the time of original presentation if subsequent imaging was used as confirmation. One-year rates for OS and LC were calculated using Kaplan-Meier estimates. Univariate analyses were then conducted using Cox regression analysis.

## Results

A total of 18 patients with a head and neck primary malignancy that were treated from 2000 to 2016 with single fraction SRS for recurrent disease were identified and analyzed in this review. Median follow-up was 16.1 months (range 0-157 months). All patients had received previous external beam radiation therapy for treatment of their primary tumor. The median interval between external beam radiotherapy and SRS was 23.7 months. The most common primary sites were nasopharynx (33.3%), parotid gland (16.7%), and sinonasal cavity (16.7%). Seven patients (38.9%) had adenoid cystic carcinoma, five patients (27.8%) had squamous cell carcinoma, and four patients (22.2%) had undifferentiated histology. The most common recurrent sites treated were the skull base (38.9%) and the cavernous sinus (22.2%). Six patients (33.3%) had distant metastases at the time of SRS. Patient characteristics are summarized in Table 1.

**Table 1:** Patient Characteristics EBRT = external beam radiation therapy, SRS = stereotactic radiosurgery, KPS = Karnofsky performance status.

Characteristics	Value (%)
Patients	N=18
Median age (yrs)	47.8
Median KPS	90
Sex	
Male	8 (44)
Female	10 (56)
Primary head and neck tumor site	
Nasopharynx	6 (33)
Sinonasal	3 (17)
Parotid	3 (17)
Hard palate	2 (11)
Unknown primary	2 (11)
Buccal mucosa	1 (6)
Infratemporal fossa	1 (6)
Histology	
Squamous cell carcinoma	5 (28)
Adenocarcinoma	2 (11)
Adenoid cystic carcinoma	7 (39)
Undifferentiated	4 (22)
Primary treatment	
Surgery + concurrent chemotherapy and EBRT	2 (11)
Concurrent chemotherapy and EBRT	5 (28)
Surgery + EBRT	11 (61)
Recurrent treatment site	
Dura	2 (11)
Nasopharynx	3 (17)
Skull base	7 (39)
Sinonasal	2 (11)
Cavernous sinus	4 (22)
SRS dose (median)	13.3 Gy
Time from EBRT to SRS (median months)	23.7

**Figure 1:**
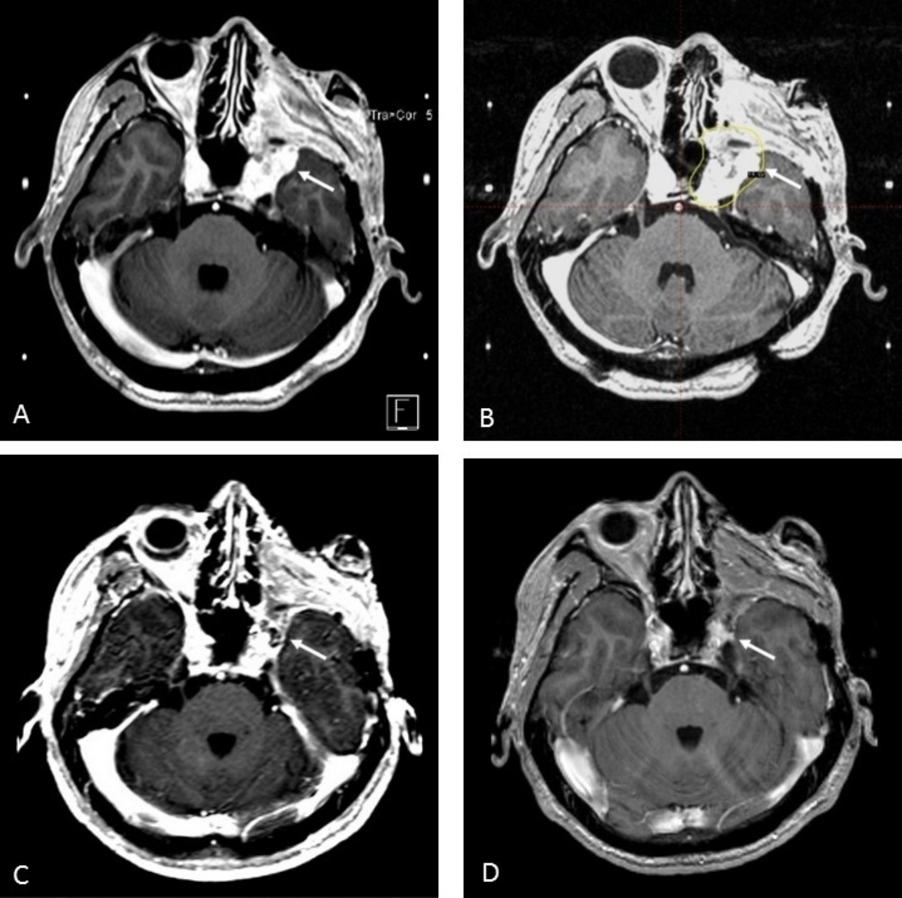
Stereotactic Radiosurgery Treatment for Recurrent Nasopharyngeal Carcinoma (A) Recurrent nasopharyngeal carcinoma in the left cavernous sinus (arrow); (B) Definitive stereotactic radiosurgery treatment plan. All gross disease covered by 14 Gy isodose line, prescribed to 50% (arrow); (C) Six-month follow-up MRI demonstrating decrease in size of soft tissue enhancement within left cavernous sinus; (D) Two-year follow up MRI showing persistent fullness in the left cavernous sinus region that has decreased in size from initial treatment and six-month scan.

The median dose prescribed was 13.3 Gy (range 10-18 Gy). Seven patients experienced PD on serial imaging and exams with a median time to progression of 13.0 months. Four patients had in-field PD, one patient had marginal PD, and two patients had out-field PD. One-year rate of LC was 52.7% (95% confidence interval [CI] 29%-72%). Three-year rate of LC was 47.4% (CI 24%-67%) (Figure 1). Upon univariate analysis, PD risk following SRS was increased for patients with a primary parotid malignancy (hazard ratio [HR] 4.24, p=0.02). The three parotid gland primary tumors reviewed in this series all had adenoid cystic histology and all suffered progression. Other primary sites, histology, recurrence site, gender, and SRS dose were not associated with LC.

**Figure 2:**
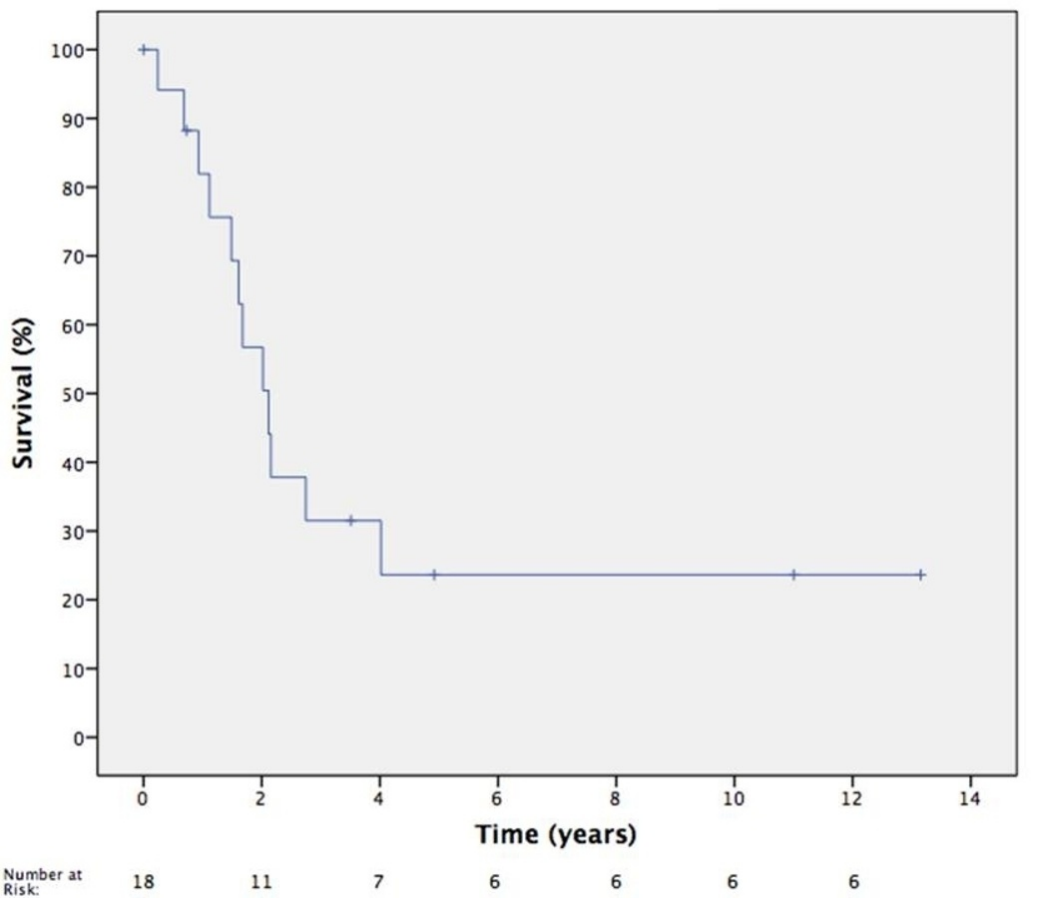
Kaplan-Meier Overall Survival Curve

The median OS was 25.4 months. One-year rate of OS was 81.9% (CI 54%-94%) and three-year rate of OS was 31.5% (CI 11%-54%) (Figure 2). OS was negatively associated with squamous cell carcinoma histology (HR 3.85, p=0.03). Karnofsky Performance Status (KPS) of ≥ 90 at the time of SRS trended toward a significant association with OS; however, it did not reach significance (p=0.07). The primary tumor site, other histologies, recurrence site, gender, age, and SRS dose were not associated with OS. One patient developed grade 2 radionecrosis following SRS to a recurrent lesion in the frontal sinus. The patient had received previous external beam radiation therapy to a dose of 68.4 Gy, six months prior to SRS (12 Gy prescribed to 50% isodose line) for an undifferentiated sinonasal primary tumor. Nineteen months following SRS, the patient presented with headaches and underwent subsequent imaging confirming radionecrosis. Oral steroids were prescribed with resolution of the patient’s symptoms. There were no other toxicities documented, including no evidence of bleeding events, soft tissue or mucosal ulceration, or osteonecrosis.  

## Discussion

Locoregional recurrence remains the primary source of failure for patients with a head and neck malignancy and treatment options remain limited [3-4]. A recent retrospective review interrogated the efficacy of salvage surgery for patients with resectable recurrences and favorable performance status [5]. Salvage surgery was associated with significant morbidity while patients with an elevated Charlson-Age Comorbidity Index, T3-T4 primary tumor, or disease-free interval of less than six months had high rates of one-year mortality. Nevertheless, salvage surgery does remain the standard of care for resectable disease in previously irradiated patients offering 25%-45% long-term disease control per a recent consensus statement [14]. Our current study involved a patient population with skull-based recurrences in anatomic locations which were deemed unresectable.

Outcomes for patients undergoing repeat external beam radiation therapy have also been previously described [15]. In one review of 74 patients receiving reirradiation for recurrent head and neck carcinomas, a two-year locoregional control rate of 64% was obtained, but with severe toxicity noted in 20% of patients and one treatment-related death.

Recurrent head and neck carcinomas have been treated with high dose rate brachytherapy with a one-year disease-specific survival of 54% and grade 3-4 late toxicity rate of 16% [16]. However, high dose rate brachytherapy is most commonly utilized for recurrences located in the oral cavity, oropharynx, or isolated nodal disease; this technique is generally not applicable at the skull base [17]. Finally, proton beam radiation therapy has been used for re-irradiation of head and neck malignancies. A multi-institutional review evaluated this approach, finding one-year locoregional failure of 25.1% with death as a competing risk. The one-year OS was 65.2%, with 9%-10% of patients having grade 3 mucositis, dysphagia, or esophagitis, and two patients having grade 5 toxicity due to treatment-related bleeding [18].

The use of SRS for recurrent head and neck carcinoma was first described by Kondziolka, et al. when they treated a recurrent nasopharyngeal tumor with a gamma knife unit [19]. This led to a prospective single arm study evaluating the use of single fraction photon based SRS for persistent or recurrent T1-T2 nasopharyngeal carcinomas. The two-year overall LC rate was 72%. Patients treated with persistent disease had a 100% control rate and those with recurrent disease had a much lower 46% control rate [20].

Fractionated SRS has been examined in 90 patients with recurrent or persistent nasopharyngeal carcinomas [11]. Severe late complications were noted in 25% of patients being treated for recurrent disease with toxicities including mucosal necrosis, brain stem necrosis, temporal lobe necrosis, and massive nasopharyngeal hemorrhage leading to death. These outcomes emphasize the difficulty of delivering a sufficient dose to achieve LC at acceptable toxicity rates in the management of recurrent head and neck malignancies.

In contrast to these reports, our study reports on the use of gamma knife radiosurgery for a variety of head and neck subsites; all patients were treated for clinically recurrent (not residual) disease. The one-year LC rate was 52.7% with one-year OS of 81.9% and median survival of 25.4 months, which are comparable to other SRS series (Table 2) [10-12, 20-22]. OS was negatively associated with squamous cell histology, but this histology was not significantly associated with local progression after SRS, highlighting the need for effective systemic therapies for such patients. Recently, cetuximab has been evaluated with concurrent stereotactic body radiation therapy (SBRT) in a prospective phase II trial for recurrent head and neck carcinomas with one-year progression-free survival of 60% and one-year OS of 40% [22]. Another study is currently evaluating the use of pembrolizumab with concurrent radiation therapy for recurrent head and neck carcinoma (Clinical Trial NCT02318771). 

**Table 2:** Stereotactic Radiosurgery Series for Treatment of Recurrent Head and Neck Malignancies LC – local control, OS – overall survival, LFFS – local failure-free survival, DSS – disease-specific survival, CR – complete response, LN – lymph node, PFS – progression-free survival, EBRT – external beam radiation therapy, SBRT – stereotactic body radiotherapy.

Author	Number of Patients	Tumor Sites included	Treatment Type	Results
Pai, et al.* *[10]	36	Recurrent nasopharynx	8-20 Gy boost after 20-60 Gy of EBRT	3 year LC– 56%, 5 year OS – 49%
Wu, et al. [11]	90	Persistent or recurrent nasopharynx	18 Gy in 3 fractions or 48 Gy in 6 fractions	Recurrent tumor 3-year LFFS – 75.1% and 3-year DSS – 45.9%
Ro, et al.[12]	36	Multiple sites of recurrent head and neck	18-40 Gy in 3-5 fractions	2-year LFFS – 52.2%, 2-year OS – 30.9%
Chua, et al. [20]	18	Persistent or recurrent nasopharynx	11-14 Gy in 1 fraction (Prescribed 80% isodose line)	Recurrent tumor 2-year LFFS – 55%
Kawaguchi, et al.[21]	22	Multiple sites of recurrent head and neck	20-42 Gy in 2-5 fractions (Prescribed 80-85% isodose line)	2-year CR – 45.5%. 2-year OS without LN metastases – 78.6%
Vargo, etal. [22]	50	Multiple sites of recurrent head and neck	40-44 Gy in 5 fractions with cetuximab (using SBRT)	1-year local PFS – 60%, 1-year OS – 40%

Furthermore, this study found that a parotid gland primary malignancy was associated with progression after SRS. All patients with a parotid gland primary malignancy suffered progression after SRS, and each had adenoid cystic histology. Even so, other primary sites of adenoid cystic tumor were included and histology as a sole factor was not associated with PD on univariate analysis. In a review of patients with recurrent salivary gland malignancies treated with chemoradiation, a 1-2 cm margin was given on the GTV to obtain a PTV. One-year and three-year rate of locoregional control was 72.2% and 51.6% respectively, emphasizing that recurrent salivary gland tumors may benefit from an additional target margin and GTV coverage alone with SRS may not be appropriate [23].

Of the seven patients who experienced PD in our study, four patients had in-field progression. Only one patient experienced an adverse event of grade 2 radionecrosis. Given this limited toxicity, more aggressive dosing with SRS may be warranted to limit in-field recurrences.

The limitations of this study include the retrospective nature, single institution experience, and small number of patients some of whom were lost to follow-up. Toxicity data was also not uniformly collected in a robust fashion. Long-term follow-up of these patients will likely also see the development of increasing late toxicities [11]. Multi-institutional efforts will help determine the proper patient selection and treatment parameters to maximize LC, maintain quality of life, and minimize toxicity for patients suffering from a head and neck disease recurrence.

## Conclusions

Single fraction SRS is an acceptable treatment option for previously irradiated patients with a recurrent head and neck primary malignancy. Given our low rates of observed toxicity and modest in-field rates of progression, dose-escalated radiosurgery should be examined to optimize LC.
